# A cross-sectional study on the self-management of asthma and asthma control among adult asthmatic patients in the Aseer region, KSA

**DOI:** 10.1038/s41598-024-67136-0

**Published:** 2024-07-12

**Authors:** Soha Makki, Ayesha Siddiqua, Bushra Ali Alqahtani, Hanan Alkhuwaylidi, Lama Alhefzi, Maram Hussain, Sarah Saeed, Waad Ahmed, Randa A. Abdelkarim, Arwa Khaled

**Affiliations:** 1 Department of Clinical Pharmacy, College of Pharmacy, King Khalid University, 61421 Abha, Kingdom of Saudi Arabia; 2 College of Pharmacy, King Khalid University, 61421 Abha, Kingdom of Saudi Arabia; 3 Department of Statistics, Faculty of Mathematical Sciences and Informatics, University of Khartoum, Khartoum, Sudan

**Keywords:** Asthma self-management, Asthma control, Knowledge, Immunology, Health care, Medical research, Risk factors, Signs and symptoms

## Abstract

Proper management of asthma is crucial for maintaining control over the disease and has a significant impact on the patient’s overall condition. The purpose of this study was to determine the extent of self-management and the level of Asthma control in the patients from the Aseer region of Saudi Arabia, as well as to investigate determinants of illness control. A study was conducted using quantitative cross-sectional methods. Researchers utilised a web-based, self-administered structured questionnaire to gather data. The questionnaire included three sections: a socio-demographic section, an Asthma Self-Management Questionnaire (ASMQ), and Asthma Control Test (ACT). An analysis using the chi-square test was conducted to determine if there was a notable connection between the socio-demographic characteristics of the participants and the level of asthma control. A total of 305 responses were collected. The average score for the Asthma Self-Management Questionnaire was 5.72, which corresponds to 40.9%. Based on the scores from the Asthma Control Test, it was found that 60 patients (20.0%) had asthma that was not under control, 94 (30.0%) had asthma that was partially controlled, and 151 (50.0%) had asthma that was well controlled. Factors such as gender, non-smoking status, and having asthma for over 10 years (*p* ˂0.05) were found to be strongly correlated with improved disease control. Significant gaps were found in patients’ awareness of the most important and critical aspects concerning the condition, medications, and preventative actions that limit asthma aggravation. To address the current situation, hospitals and clinics must make substantial efforts.

## Introduction

Asthma is a chronic respiratory condition characterized by airway inflammation and hyperreactivity. Effective self-management is crucial for maintaining control and reducing the frequency and severity of asthma attacks. Various studies and literature have explored the relationship between self-management practices and asthma control^1,2^. An asthma diagnosis is confirmed with spirometry and pulmonary function test, where spirometry, which measures how much air a patient can expel in one forced breath, is a noninvasive test used for diagnosing and monitoring a variety of lung disorders^3,4^. Many factors may trigger asthma, such as weather changes, seasonal allergies, air pollution, smoking, medication like aspirin, highlands, pets, genetics, Dust, perfumes, and cosmetics. In addition, commodities that may affect the severity of asthma include infections, allergies, obesity, anxiety, stress, depression, and obstructive sleep apnea^5,6^. In Saudi Arabia, more than 2 million are diagnosed with asthma, and 10.6% of asthmatic patients use medications to control the symptoms^7^. Many studies show the prevalence of uncontrolled asthma patients in Saudi Arabia is about 77.0% in females and 58.9% in males. The prevalence of asthma in the Aseer region was found to be high (19.2%) compared with other areas of Saudi Arabia (9%)^7^. Asthma has no cure; its symptoms are managed to prevent complications and reduce morbidity and mortality. “Self-management” refers to “the ability of a patient to control the symptoms and improve the quality of life.” Patient education about asthma disease and the correct use of medication, especially how to use the inhaler, and ensuring patient adherence with complete counselling from the pharmacist about the treatment plan is vital to improve self-management and minimize hospitalization. Many factors may have contributed to the uncontrolled asthma symptoms, such as incorrect diagnosis, poor knowledge demonstrated by physician related to asthma management in addition to the lack of appropriate explanation on the use of the medication or total management, or the doctor did not provide the patient enough time to explain to the patient how to use medication or complete management of the patient condition.

The previous study in Taif, KSA, in 2016 demonstrated that the male sex exhibited better control. The asthma control rate was low, and patients had significant gaps in essential elements of asthma prevention strategies and the use of medications that are critical for the self-management of the disease^8^. Previous studies were carried out in Wisconsin during 2012–2016. During the period of the study, the majority of individuals did not have well-controlled asthma. There were substantial disparities across asthma control groups about the proportion of individuals who had routine asthma checks. Moreover, there was a considerable difference in the percentage of asthma control at admission. People with asthma with a yearly household income of $50,000 or greater have better asthma control^9^. Another study in Vietnam revealed that adult patients’ asthma self-management knowledge is limited and correlated with their education degree. A positive correlation was established between self-management and asthma control, suggesting that the greater the level of asthma control, the greater the asthma self-management knowledge (and vice versa). There is a need to increase asthma self-management knowledge among adult patients in Vietnam, particularly those with a lower level of education, via their physicians or other sources^3^.

Torchyan’s study revealed that a variety of characteristics, including stress, occupation, and obesity, had a differential relationship with uncontrolled asthma between men and women in Saudi Arabia, which could provide further knowledge on how to enhance asthma control, particularly in women^6^.

Also, in a previous study of the Aseer region, southwestern Saudi Arabia, one out of every five adults had bronchial asthma. The following factors are significantly associated with bronchial asthma in adults. They live in low‐altitude areas and the countryside, take analgesics, live near busy streets, have cats at home, and are 55–64 years old. There is an urgent need to improve the asthma self-management program at the primary health care level, especially in rural and low-altitude areas, and more focus should be placed on the elderly. In addition, there is a need to improve community health promotion programs that address bronchial asthma modifiable factors such as analgesic abuse, outdoor pollution, and cats in and around the houses of predisposed individuals. It is also recommended to carry out epidemiological studies and national studies in these areas^5^.

Previous research demonstrates a substantial correlation between asthma self-management and asthma control; consequently, it is necessary to define asthmatic patients’ self-management expertise. The purpose of this study was to determine the extent of self-management and the level of Asthma control in the patients from the Aseer region of Saudi Arabia, as well as to investigate determinants of illness control. Self-management of asthma entails healthcare practitioners assisting, educating, and training individuals with asthma so they can learn how to manage their symptoms independently. However, there is limited evidence regarding the level of assistance patients require from their healthcare team.

## Methods

### Study design, setting, and population

The current research adopted an observational cross-sectional study employing a well-structured web-based questionnaire. The study was conducted in Saudi Arabia from April 2022 to June 2022. The study comprised 305 outpatients patients at Aseer Hospital. Data were collected through a web-based questionnaire method. The data collection instrument consisted of three sections: 1. Socio-demographic Section. 2. Asthma Self-Management Questionnaire (ASMQ)^10^. 3. Asthma Control Test (ACT)^11^.

### Study variables

Age, gender, residence, marital status, smoking status, attending formal asthma education, duration of asthma, and employment.

### Inclusion criteria

This study included participants older than 18 diagnosed with asthma at least six months before data collection.

### Exclusion criteria

Participants with a significant medical condition or mobility-limiting co-morbidity were excluded from this study, as were those with cognitive deficits, difficulty in speaking, pregnant or lactating women, and patients with chronic obstructive pulmonary disease or an illness with symptoms similar to asthma.

### Sample size and sampling technique

The survey was conducted on the outpatients. When the patients visited the outpatient clinic they were requested to fill the questionnaire. Non-probability convenience sampling method was used to recruit the participants. The researchers approached the target participants, and the interested participants were asked the questions from the survey by the researchers. If there was any ambiguity in understanding the questions, the researchers helped the participants.

### Sample size calculation

The Raosoft program was used to estimate the sample size using a margin of error of 5% and a 95% confidence interval^12^. The estimated final sample size calculated was 305. 377 patients were approached but only 305 patients responded, remaining were not willing to participate, the total population used to calculate the sample size was 20,000.

### Data collection and statistical analysis

Before completing the questionnaire, the participants could introduce themselves and learn more about the study’s purpose. The participants were assured that their responses would be kept anonymous for a valid response.

The tool used for data collection was a web-based structured knowledge questionnaire comprised of multiple-choice questions. The purpose of the study was clearly stated in the questionnaire. The questionnaire consists of three main parts: the first includes demographic variables, the second contains questions regarding responses to the Asthma Control Test, and the third part focuses on asthma Self-management knowledge. Each response was given a score of one, and all the scores were summed to get a raw score that ranged from 0 to 16. Then, the raw score was transformed to a scale of 0 to 100, and the final score was reported. A higher score represented participants with more excellent knowledge of self-management of asthma. A total of 305 individuals completed the questionnaire and were included in the current study.

A Statistical Package for Social Sciences version 20 (SPSS Inc., Chicago, IL, USA) was used to analyze the data (SPSS). Two expert statisticians did the analysis. Descriptive and inferential statistics were used for the data analysis. The *p*-value of less than 0.05 was considered significant.

### Ethical approval

Ethical approval for this study was obtained from the “Ethical Committee of King Khalid University, Ethical approval NO: ECM#2021-5910”. This study complies with the Declaration of Helsinki and was performed after obtaining ethics committee approval. An informed consent form was available on the first page of the questionnaire, stating that participation is voluntary, that confidentiality is guaranteed, and that all information will be used solely for research reasons.

## Results

### Demographic characteristics of participants

In total, 305 patients were included, with 167 (55%) females. Most patients in the community were between the ages of 45 and 60. 90% of patients reported attending formal asthma education programs. Table 1 displays socio-demographic information about research participants.

**Table 1:** Participants’ socio-demographic data.

Demographic		N	%
Gender	Female	167	55%
	Male	138	45%
Age in years	18–25 years	83	27%
	25–45 years	82	27%
	45–60 years	122	40%
	More than 60 years	18	6%
Residence	Outside the town	117	38%
	Town	188	62%
Educational level	Illiterate	10	3%
	Primary school	10	3%
	Secondary school	82	27%
	Graduated	174	57%
	Post-graduation	29	10%
Marital status	Married	180	59%
	Single	125	41%
Employment	Employed	149	49%
	Unemployed	156	51%
Smoking status	Ex-smoker	60	20%
	Non-smoker	195	64%
	Smoker	50	16%
Duration of asthma	0–10 years	118	39%
	More than 10 years	187	61%
Attending formal asthma education	No	30	10%
	Yes	275	90%
Total		305	100%

N, number of participants; %, percentage.

### Responses and total score to Asthma Self-Management Questionnaire

The mean score for (ASMQ) was 40.9%. Table 2 demonstrates the patients’ correct responses to the self-management Questionnaire. Overall, 72% of patients answered that they should hold their breath after taking the dose, which means they could follow instructions on how to use their inhalers. However, a lower percentage was observed when patients were asked about the knowledge that exercise helps improve breathing capacity.

**Table 2:** Patient correct answers to the self-management questionnaire (N = 305).

Question	Answers	N	%
A primary method to prevent asthma flare-ups is to…	Get a flu vaccine	104	34%
Take the prescribed two puffs of your inhaler two times a day	It’s not the same as any other regimen	129	42%
If you are not having asthma symptoms…	You should still avoid triggers	167	55%
Maintenance medicines…	help prevent future symptoms	116	38%
The correct way to use peak flow meter is to…	Take a deep breath, then blow into the mouthpiece as fast as you can	95	31%
Rescue medicines…	should not be taken more than three or four times a day	88	29%
When using your inhaler, you should…	inhale slowly	96	31%
After you have used your inhaler, you should…	Hold your breath for several seconds	219	72%
If you are having symptoms and don’t know why, the first thing you should do is…	Change your immediate environment	141	46%
Taking more rescue medicines than prescribed	may mean you need more maintenance medicine	90	30%
The benefit of using a peak flow meter every day is…	You can detect small changes in lung function even before symptoms start	132	43%
For people with asthma, exercise…	can help improve breathing capacity	61	20%
Asthma can be cured by…	There is no known cure for asthma	85	28%
Asthma flare-ups.…	can occur when several minor triggers come together	120	39%
If you are prescribed a seven-day course of steroid pills…	You should finish the prescription even if you feel better after several doses	200	66%
Which of the following can help control asthma?	All of the above	129	42%

N, number of participants; %, percentage.

### Asthma control

The Response to the Asthma Control Test is shown in Table 3. According to the scores, 60 patients (20.0%) had uncontrolled asthma, 94 (30.0%) had partially controlled asthma, and 151 (50.0%) had controlled asthma.

**Table 3:** Response to the asthma control test.

During the past 4-weeks, how often have you had shortness of breath:									
3–6 times a week		More than once a day		Not at all		Once a day		Once or twice a week	
N	%	N	%	N	%	N	%	N	%
76	25%	11	4%	62	20%	40	13%	116	38%

During the past 4- weeks how often did your asthma symptoms wake you up at night or earlier than usual in the morning:									
2–3 nights a week		4 or more night a week		Not at all		Once a Week		Once or twice a month	
N	%	N	%	N	%	N	%	N	%
47	15%	13	4%	76	25%	74	24%	95	31%

In the past 4 weeks, how much of the time did your asthma keep you from getting as much done at work, at school, at home:									
A little of the time		All the time		Most of the time		Not at all		Some of the time	
N	%	N	%	N	%	N	%	N	%
120	39%	5	2%	39	13%	66	22%	75	25%

During the past 4 weeks, how often have you used your rescue inhaler or nebulizer medication									
1–2 times per day		2–3 times per week		3 or more times per day		Not at all		Once a week or less	
N	%	N	%	N	%	N	%	N	%
43	14%	82	27%	11	4%	52	17%	117	38%

How would you rate your asthma control during the past 4 weeks:									
Completely controlled		Not controlled at all		Poorly controlled		Somewhat controlled		Well controlled	
N	%	N	%	N	%	N	%	N	%
50	16%	41	13%	19	6%	94	31%	101	33%

N, frequency; %, percentage.

### Predictors of asthma control

The results showed that regardless of gender, non-smoker, and more than 10 years duration of asthma (*P* ˂ 0.05), these variables were highly associated with better disease control, as described in Table 4.

**Table 4:** Asthma control predictors.

		How would you rate your asthma control during the past four weeks?						*p*-value
		Uncontrolled		Partially controlled		Controlled		
		N	%	N	%	N	%	
Gender	Female	33	55%	48	51%	86	57%	0.666
	Male	27	45%	46	49%	65	43%	
Age in years	18–25 years	24	40%	21	22%	38	25%	0.19
	25–45 years	10	17%	27	29%	45	30%	
	45–60 years	24	40%	39	41%	59	39%	
	˃ 60 years	2	3%	7	7%	9	6%	
Residence	Outside the town	23	38%	37	39%	57	38%	0.969
	Town	37	62%	57	61%	94	62%	
Educational level	Graduated	29	48%	53	56%	92	61%	0.188
	Illiterate	1	2%	2	2%	7	5%	
	Post-graduation	4	7%	8	9%	17	11%	
	Primary school	4	7%	2	2%	4	3%	
	Secondary school	22	37%	29	31%	31	21%	
Marital status	Married	30	50%	54	57%	96	64%	0.182
	Single	30	50%	40	43%	55	36%	
Employment	Employed	22	37%	49	52%	78	52%	0.108
	Unemployed	38	63%	45	48%	73	48%	
Smoking status	Ex-smoker	16	27%	21	22%	23	15%	0.012
	Non-smoker	35	58%	50	53%	110	73%	
	Smoker	9	15%	23	24%	18	12%	
Duration of asthma	0–10 years	30	50%	40	43%	48	32%	0.032
	˃ 10 years	30	50%	54	57%	103	68%	

*p*-value is significant at < 0.05; N, number of participants; %, percentage.

## Discussion

Asthma is a chronic respiratory condition characterized by airway inflammation and hyperreactivity. Effective self-management is crucial for maintaining control and reducing the frequency and severity of asthma attacks. The current study’s demographic data revealed that more than half of the patients recruited were females. According to the Nicolai et al. cohort’s study, males are likelier than females to suffer from asthma; however, the gender ratio is reversed in adults. It is unclear when this transformation occurs or by what mechanism^13^. Most patients in this study were between 45 and 60.

Asthma in the elderly might be challenging to diagnose due to changes in clinical and functional characteristics^14^ elderly experience daytime symptoms, associated with a poor prognosis leading to an eventual loss of functions^15^ patients aged between 45–60 years old live in town In low- and middle-income countries (LMICs), urbanization has been linked to temporal and geographical changes in asthma prevalence. However, little is known about the mechanisms that link urbanization with asthma, which may be explained by the empirical approaches employed to evaluate the urbanization-asthma relationship^16,17^, the recruited patients live in a high-altitude area that might contribute to the occurrence and the worsening of the patient’s symptoms. Similar studies were conducted and had similar findings^18^ a meager percentage of the recruited patients were actively smoking, and a higher percentage of patients were non-smokers. In recent studies, tobacco smoking has been linked to more severe asthma symptoms, a faster deterioration in lung function, and worse responses to corticosteroids^19^. This study also agrees with a recent study conducted in Saudi Arabia, which indicated a decrease in the quality of life of asthmatic male patients who used daily smoking and women who had a household member who smoked inside the house^20^.

In general, recruited patient responses to the Asthma Self-Management Questionnaire uncovered significant gaps in patients’ knowledge about asthma and poor understanding of the treatment and the approaches for preventing asthma attacks. The main evident issue was the poor inhaler technique knowledge on how to use the inhaler, as the patient failed to repeat simple usage instructions. The current study sheds light on the poor disease control caused by a lack of healthcare training and counselling during patient appointments/visits. This outcome has been, however, observed in several studies^21,22^.

Most patients did not know the difference between the medications that should be used to relieve the symptoms of acute asthma attacks and long-term maintenance therapy. Also, most patients did not know they would require maintenance medicines if they were using short-acting reliever medications. The final result of poor patient understanding of their disease state and knowledge of the treatment options directly impacted the absolute compliance to therapy^23^ Almost 35% of patients in the current study demonstrated good knowledge regarding the role of flu vaccination in reducing the occurrence of asthma flare-ups; many studies reported a correlation between viral infections and asthma exacerbation^24,25^. However, only 28% of patients knew that asthma is not a curable disease; a lack of knowledge of this fact might have contributed to the poor compliance as many patients stop their medication once the asthma attack has subsided or if they don’t suffer from symptoms. A fascinating finding noted in this study is that approximately 50% of the patients considered that their disease was controlled or partially controlled during the previous four weeks. This finding proves that patients are under the impression that their condition is under control and that it is all attributed to a lack of knowledge to correlate symptoms with poor control.

Various studies and literature have explored the relationship between self-management practices and asthma control. A study published by NCBI found that higher levels of asthma control (measured by ACT or GINA criteria) correlated with better self-management knowledge (measured by ASMQ scores). Patients with better self-management knowledge had better asthma control. It was noted that the level of knowledge on asthma self-management was generally low among patients. This lack of knowledge significantly affected their level of asthma control^26^. A study published in Nature discussed various barriers and facilitators to effective self-management in asthma. Despite being an established method for controlling asthma, the promotion, uptake, and use of self-management practices vary widely^27^. Research from the Journal of Managed Care & Specialty Pharmacy (JMCP) showed that patients who received self-management support services from community pharmacists had better symptom control and lower severity^28^. A randomized controlled trial published in the Health Education Research journal concluded that an asthma self-management model significantly improved the quality of life for asthma patients, further emphasizing the importance of self-management in asthma control^29^.

Self-management education, which includes personalized action plans, has been shown to significantly improve asthma control. According to a Cochrane review, self-management education that includes a written action plan and regular medical review is associated with reduced hospitalizations, emergency room visits, and days off work or school due to asthma^30^. Empowering patients through education and training enables them to recognize and manage symptoms, use medications appropriately, and avoid triggers. This empowerment leads to improved asthma outcomes. For example, a study by Gibson et al. demonstrated that patients who received self-management education had better asthma control and quality of life compared to those who received usual care^30^. Adherence to prescribed medication regimens, especially inhaled corticosteroids, is a cornerstone of asthma self-management. Studies have shown that poor adherence is a major factor contributing to poor asthma control. A study by Bender et al. found that patients with higher adherence to controller medications had better asthma control and fewer exacerbations^31^. The development of mobile health applications has opened new opportunities for asthma self-management. mHealth apps can include prescription reminders, symptom tracking, and instructional resources. Marcano Belisario et al. conducted a systematic study that emphasised the potential of mHealth interventions to improve medication adherence and asthma control^32^. Telemedicine has emerged as an effective asthma care method, especially during the COVID-19 pandemic. Virtual consultations enable continual monitoring and support, which can improve self-management habits. Studies have demonstrated that telemedicine can enhance asthma control and patient satisfaction^33^.

This study has its own limitations. The sample size is small so the study cannot be generalized. While this study investigated asthma control and self-management, it has numerous limitations due to the lack of precise data on some variables. The severity of asthma in individuals was not specifically specified, which may restrict the generalizability of our findings to diverse asthma phenotypes and illness severities. Additionally, patients’ allergy status, including allergen sensitivities, para-clinical findings like skin test results and spirometry readings prevented a complete knowledge of asthma control’s physiological and immunological aspects. Our results may be limited by the absence of these critical variables, highlighting the need for future research to assess asthma severity, allergic status, and para-clinical findings to better understand their effects on asthma control and management as well as to estimate the association between self-management and asthma control.

## Conclusion and recommendations

Significant gaps were found in patients’ awareness of the most important and critical aspects concerning the condition, medications, and preventative actions that limit asthma aggravation. To address the current situation, hospitals and clinics must make substantial efforts. Effective self-management is critical to maintaining optimal asthma control. The results showed that regardless of gender, non-smoker, and more than 10 years duration of asthma, these variables were highly associated with better disease control.

Education, medication adherence, regular monitoring, and personalized action plans are all important factors that lead to better outcomes. Addressing hurdles such as health literacy and socioeconomic status, as well as utilizing technology, can help to improve self-management behaviors. Continued research and personalized interventions are required to help patients effectively manage their asthma.

## Data Availability

The datasets used and/or analyzed during the current study available from the corresponding author on reasonable request.
